# A Prognostic Model Based on Nine DNA Methylation-Driven Genes Predicts Overall Survival for Colorectal Cancer

**DOI:** 10.3389/fgene.2021.779383

**Published:** 2022-01-21

**Authors:** Zhongsheng Feng, Zhanju Liu, Kangsheng Peng, Wei Wu

**Affiliations:** Department of Gastroenterology, Shanghai Tenth People’s Hospital, Tongji University, Shanghai, China

**Keywords:** nomogram, risk score, colorectal cancer, DNA methylation, prognosis

## Abstract

**Background:** Colorectal cancer (CRC) is the third most frequently diagnosed malignancy and the fourth leading cause of cancer-related death among common tumors in the world. We aimed to establish and validate a risk assessment model to predict overall survival (OS) for the CRC patients.

**Methods:** DNA methylation-driven genes were identified by integrating DNA methylation profile and transcriptome data from The Cancer Genome Atlas (TCGA) CRC cohort. Then, a risk score model was built based on LASSO, univariable Cox and multivariable Cox regression analysis. After analyzing the clinicopathological factors, a nomogram was constructed and assessed. Another cohort from GEO was used for external validation. Afterward, the molecular and immune characteristics in the two risk score groups were analyzed.

**Results:** In total, 705 methylation-driven genes were identified. Based on the LASSO and Cox regression analyses, nine genes, i.e., *LINC01555*, *GSTM1*, *HSPA1A*, *VWDE*, *MAGEA12*, *ARHGAP*, *PTPRD*, *ABHD12B* and *TMEM88*, were selected for the development of a risk score model. The Kaplan–Meier curve indicated that patients in the low-risk group had considerably better OS (*P* = 2e-08). The verification performed in subgroups demonstrated the validity of the model. Then, we established an OS-associated nomogram that included the risk score and significant clinicopathological factors. The concordance index of the nomogram was 0.81. A comprehensive molecular and immune characteristics analysis showed that the high-risk group was associated with tumor invasion, infiltration of immune cells executing pro-tumor suppression (such as myeloid-derived suppressor cells, regulatory T cells, immature dendritic cells) and higher expression of common inhibitory checkpoint molecules (ICPs).

**Conclusion:** Our nine-gene associated risk assessment model is a promising signature to distinguish the prognosis for CRC patients. It is expected to serve as a predictive tool with high sensitivity and specificity for individualized prediction of OS in the patients with CRC.

## Introduction

Colorectal cancer (CRC) is the third most frequently diagnosed malignancy and the fourth leading cause of cancer-related death among of those common tumors in the world (Arnold et al., 2017). The morbidity of CRC worldwide is expected to increase to more than two million new cases by 2035 (Dekker et al., 2019). Curative surgical resection and chemotherapy are still the main treatment (Dekker et al., 2019). Although great progress has been made in the diagnosis and treatment of CRC in recent years, the prognosis of colorectal cancer remains unsatisfactory. Currently, the prognosis assessment of colorectal cancer staging based on tumor-node-metastases (TNM) classification system is insufficient for prognostic estimation, which limited the clinical decision-making (Compton, 2007). The exploration of effective biomarkers for early assessment is an important preventive measure to improve the prognosis of CRC.

Pursuing predictors of colorectal cancer, a growing number of studies have identified valuable biomarkers, such as Integrin beta-4 (*ITGB4*) (Li et al., 2019), Placenta-specific protein 1 (*PLAC1*) (Ren et al., 2020), some miRNAs and lncRNAs(Hibner et al., 2018; Dastmalchi et al., 2020). However, due to lack of sufficient sensitivity and specificity, few biomarkers have been successfully applied in clinical practice.

DNA methylation represents an important epigenetic modification that regulates gene expression (Dor and Cedar, 2018). In humans, DNA methylation mainly occurs on CpG dinucleotides. CRC develops through cumulative genetic and epigenetic changes in the precursor lesions (such as adenomas and serrated lesions) (Jung et al., 2020). Currently, hypermethylation in promoter regions of some important tumor suppressor genes that cause gene expression inhibition has been identified in colorectal cancer cells (de Vallière et al., 2015), such as Cyclin-dependent kinase inhibitor 2A (*CDKN2A*) (Bihl et al., 2012), DNA mismatch repair protein Mlh1 (*MLH1*) (Cunningham et al., 1998), and Adenomatous polyposis coli protein (*APC*) (Liang et al., 2017). The aberrant methylation may be a key event in the progression of colorectal cancer. Therefore, deregulated DNA methylation and corresponding gene expression changes might be used as biomarkers to play a prospective role in the early diagnosis, prognosis and clinical decision-making for CRC, which is worthy of further study.

By integrating DNA methylation and mRNA expression profile data, we aimed to screen out CRC-associated DNA methylation-driven genes and evaluate the potential of these genes expression to predict CRC prognosis. We identified a prognosis-related gene panel made up of nine genes and developed a risk score model. Then we established a nomogram by combining the nine-gene signature and some significant clinicopathologic factors to predict overall survival (OS) in patients with CRC. The model was validated in one Gene Expression Omnibus (GEO) cohort. Furthermore, we characterized the molecular and immune profile of nine-gene signature. These results showed that the nine-gene risk model was a promising prognostic biomarker for CRC patients.

## Materials and Methods

### Data Acquisition and Preprocessing

The available RNA-seq transcriptome data, gene mutation information and clinicopathological information were directly downloaded from the TCGA database (https://portal.gdc.cancer.gov/). There were 476 samples with gene transcriptome data (41 normal and 435 tumor), 347 patients with mutation information, and 400 patients with available survival data. TCGA-Assembler 2 (Wei et al., 2018) was used to obtain Level 3 methylation data (37 normal and 279 tumor) from the TCGA Methylation 450 k Bead chip. Then the function CalculateSingleValueMethylationData of TCGA-Assembler two was used to calculate the average methylation level of each gene. The methylation levels of genes were scored using β values ranging from 0 to 1 (unmethylated to totally methylated). The external validation cohort was from a GEO dataset of gene expression arrays [GSE39582 (N = 550)] (https://www.ncbi.nlm.nih.gov/geo/query/acc.cgi?acc=GSE39582). “Deseq2” package of R was used to normalize the raw RNA-seq data and identify differentially expressed genes (DEGs) between normal and cancer groups (Anders and Huber, 2010).

### Identification of DNA Methylation-Driven Genes

Through the integration of gene expression and DNA methylation datasets, “MethylMix” package of R software was employed to recognize DNA methylation-driven genes (Gevaert, 2015). There are three steps. Firstly, a linear regression model was built to estimate the association between gene methylation and gene expression, and genes with a significant inverse relationship (*p* value < 0.01) were selected. Such genes were defined as transcriptionally predictive genes. Secondly, a beta mixture model was created to determine the methylation states of each gene. Thirdly, the Wilcoxon rank tests were computed to compare the methylation levels between each methylation state and normal tissue samples. Differential genes were collected. Lastly, genes that were both transcriptionally predictive and differential were selected as DNA methylation-driven genes.

### Function Enrichment and Pathway Analysis

Metascape (Zhou et al., 2019) was used to perform GO Biological Processes enrichment and KEGG pathway analysis of the genes identified by MethylMix. Only terms with *p* < 0.01, a minimum overlap of 3 and an enrichment factor >1.5 were considered significant.

### Feature Selection and Building the Predictive Signature

Initially, the genes identified by MethylMix were applied to a univariable Cox regression and a LASSO Cox regression. In univariable Cox regression analysis, genes with *p* < 0.05 were selected. In LASSO regression, genes were screened 1,000 times, and if specific genes were detected more than 700 times, they were regarded as candidates. The intersection of univariable Cox regression and LASSO regression (nine genes) were brought into a multivariable Cox regression analysis. The linear combination of the regression coefficient derived from the multivariable Cox regression model (β) multiplied by its mRNA level generated a prognostic risk score with nine genes.

### Development and Validation of the Risk Score Model

Using X-tile software (Camp et al., 2004) to determine the optimal cut-off value, we divided patients into low- and high-risk groups. Then the Kaplan–Meier curves and time-dependent receiver operating characteristic curve (tdROC) analysis were performed to evaluate the predictive accuracy of the nine-gene signature risk model for OS. The survivalROC function in “survivalROC” package of R was used to draw tdROC and the span argument was set as 0.0657. A subgroup analysis was performed by dividing the patients based on clinicopathological characteristics.

### Development and Assessment of the Nomogram in the TCGA Dataset

The significance of the risk score model and other traditional clinicopathological characteristics to predict OS was evaluated by univariable Cox regression analysis. Then, we used multivariable Cox regression analysis and stepwise regression method to distinguish significant predictive factors, from which we built a nomogram predictive model. The concordance index (C-index) was computed to evaluate the accuracy of the nomogram. The prognostic risk value of each patient was calculated using the nomogram and tdROC curve analysis was used to further validate the predictive performance of the nomogram. The span argument of survivalROC function was set as 0.0657. The survival estimation and ROC curve of patients above were analyzed with the Kaplan–Meier method by “survival”, “survivalROC” and “plotROC” packages of R.

### External Validation of the Nomogram

In the validation phase, we verified the nomogram by using another CRC dataset, GSE39582 in the GEO. The discrimination of model was assessed by the AUC of tdROC and concordance index. The span argument of survivalROC function was set as 0.0616. The calibration of model was visualized in calibration plot and quantified by the slope of the calibration line.

### Weighted Gene Correlation Network Analysis

All robustly expressed genes (nearly 18,000 genes) were used for WGCNA analysis. The analysis was performed as described previously (Langfelder and Horvath, 2008). Briefly, we first screened the best soft threshold by “WGCNA” package (Langfelder and Horvath, 2008) of R to ensure a scale-free network. In the co-expression network, genes with high absolute correlations were clustered into the same module. Furthermore, module eigengenes (MEs) were defined as the first principal component of each gene module and the expression of MEs was considered as a representative of all genes in a given module. The correlation between MEs and clinical trait was calculated to identify the clinical key module. GO and KEGG enrichment analysis of the key module resulted from WGCNA were performed by Metascape. ModuleMembership (MM) was defined as the Pearson’s correlation between gene expression and ME. Gene significance (GS) was defined as the Pearson’s correlation between gene expression and certain clinical trait. The cut-off criteria was set as |MM|>0.8, |GS|>0.3 to identify hub genes with high connectivity in the clinical key module. Gene Expression Profiling Interactive Analysis (GEPIA) database (Tang et al., 2017) (http://gepia.cancer-pku.cn/) is an interactive website application that includes the transcriptome data in TCGA and GTEx projects and integrate them in a widely accepted process. We inputted the hub genes into the GEPIA and validated these hub genes.

### Gene Set Enrichment Analysis

Differential expression analysis was first performed on all genes to analyze the samples with high and low nine-gene score using “DESeq2” package of R. Enrichment analysis to determine the signaling pathways in which the differentially expressed genes are involved was then carried out by using GSEA method based on the GO Biological Processes and Hallmark gene sets with the “clusterProfiler” (Yu et al., 2012) package of R. A false discovery rate (FDR) less than 0.25 and an absolute value of the normalized enrichment score (NES) greater than 1 were defined as the cutoff criteria.

### Gene Mutation Analysis

In the gene mutation analysis, information on genetic alterations was directly obtained from the TCGA database portal, and the quantity and quality of gene mutations were analyzed by using the “Maftools” package (Mayakonda et al., 2018) of R.

### Analysis of Tumor-Infiltrating Immune Cells Characteristics

The ssGSEA by “GSVA” package (Hänzelmann et al., 2013) of R was introduced to quantify the relative infiltration of 28 immune cell types in the tumor microenvironment. Feature gene sets for each immune cell type were obtained from a recent publication (Charoentong et al., 2017). The gene sets include 782 genes for predicting the abundance of 28 TIICs in individual tissue samples. The following 28 types of immune cells include: activated B cells (Ba), activated CD4^+^ T cells (CD4^+^ Ta), activated CD8^+^ T cells (CD8^+^ Ta), activated dendritic cells (DCa), CD56bright natural killer cells (CD56^+^ NK), CD56dim natural killer cells (CD56^−^ NK), central memory CD4^+^ T cells (CD4^+^ Tcm), central memory CD8^+^ T cells (CD8^+^ Tcm), effector memory CD4^+^ T cells (CD4^+^ Tem), effector memory CD8^+^ T cells (CD8^+^ Tem), eosinophils, gamma delta T cells (γδT), immature B cells (Bim), immature dendritic cells (DCim), mast cells, myeloid-derived suppressor cells (MDSC), memory B cells (Bm), monocytes, natural killer cells (NK), natural killer T cells (NK T), neutrophils, plasmacytoid dendritic cells (DCp), macrophages, regulatory T cells (Tregs), follicular helper T cells (Tfh), type-1 T helper cells (Th1), type-17 T helper cells (Th17), and type-2 T helper cells (Th2). The relative abundance of each immune cell type was represented by an enrichment score in ssGSEA analysis. The ssGSEA score was normalized to unity distribution, for which zero is the minimal and one is the maximal score for each immune cell type.

### Statistical Analysis

The bilateral log-rank test was used in the Kaplan–Meier survival examination to compare OS between different subgroups. Wilcoxon rank-sum test was used to evaluate the differences between two groups. All statistical analyses were conducted with R software (version 4.0.3). All statistical tests were two-sided, and *p* values less than 0.05 were considered statistically significant.

## Results

### TCGA Data Acquisition and Identification of DNA Methylation-Driven Genes

**FIGURE 1:**
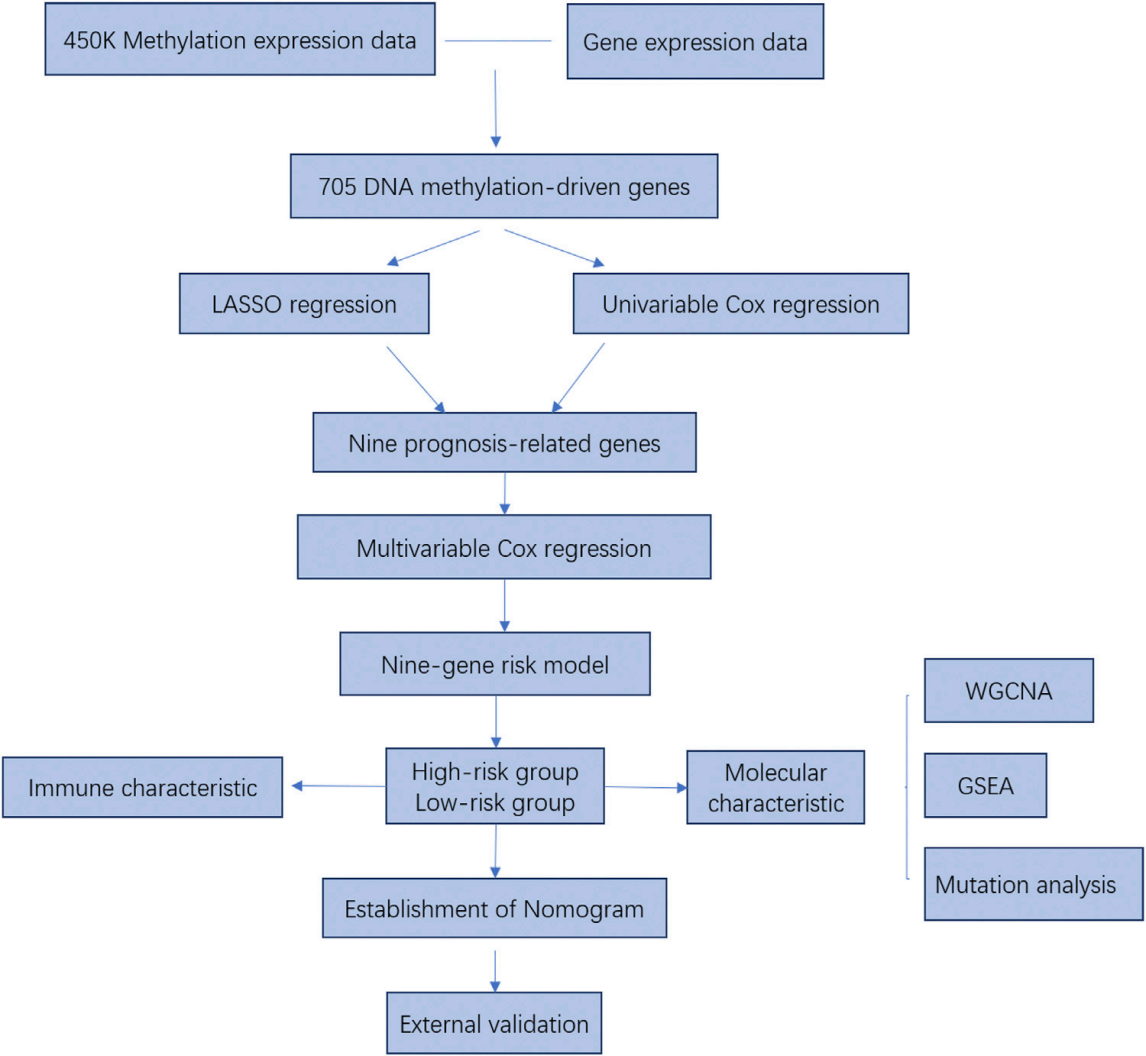
The flow chart of the study design and analysis.

**FIGURE 2:**
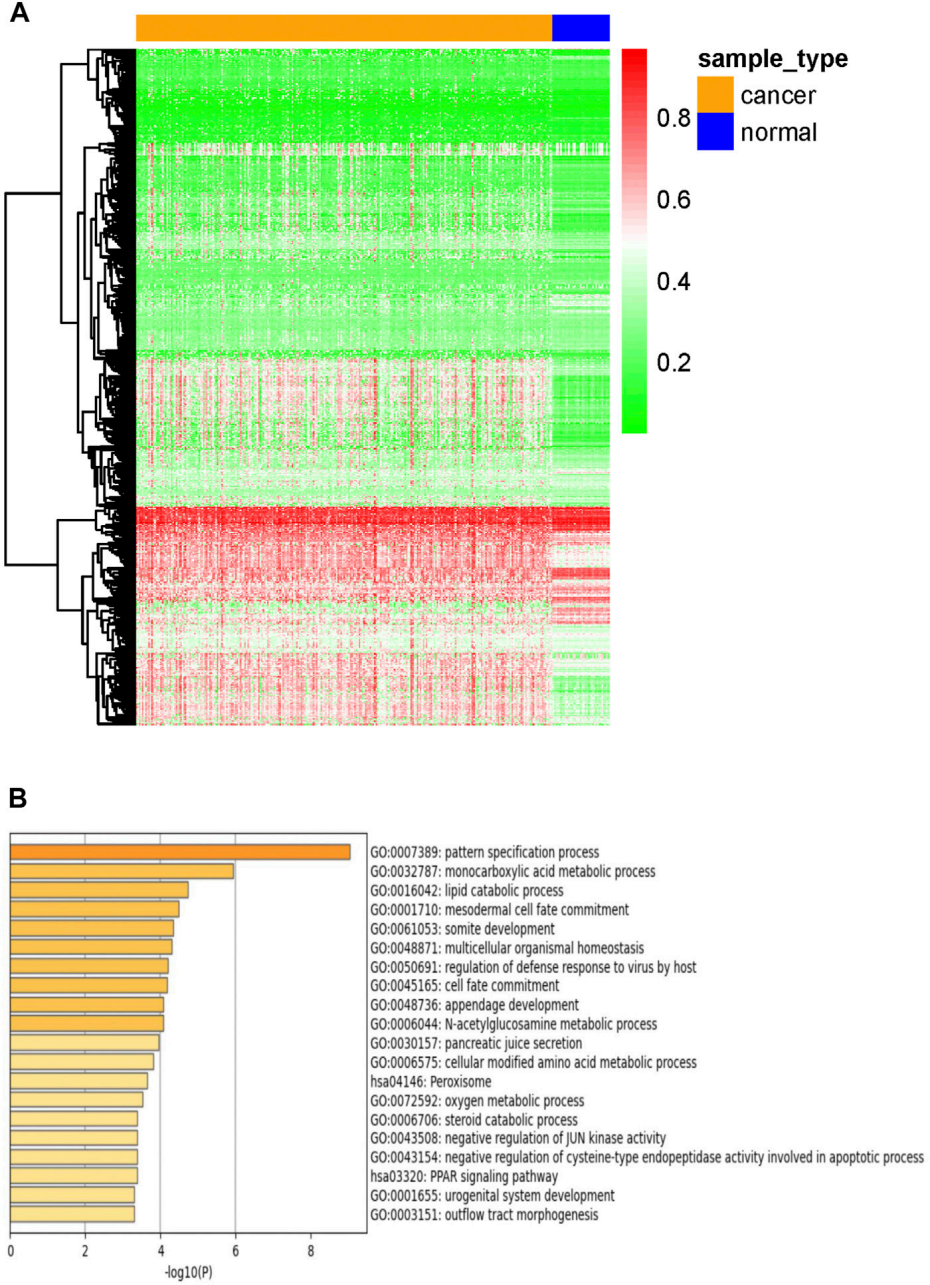
Candidate DNA methylation-driven genes screened by MethylMix. **(A)** Heatmap of the candidate DNA methylation-driven genes (*n* = 705) in cancer and normal tissues. **(B)** GO/KEGG analysis of DNA Methylation-driven genes.

The study flowchart describing the process is shown in Figure 1. To identify DNA methylation-driven genes in CRC, we performed MethylMix analysis on 273 tumor samples and 37 normal tissue samples. A total of 705 genes were identified and a mixture model of each gene was constructed (Supplementary Figure S1). Their methylation levels were visualized by heat map (Figure 2A). Metascape analysis showed the first 20 clusters of enriched sets (Figure 2B). For biological process (BP), these genes showed enrichment in pattern specification process, lipid catabolic process, multicellular organismal homeostasis, cell fate commitment and so on. The KEGG pathway data were enriched in peroxisome and PPAR signaling pathway.

### Establishment of a Prognostic Risk Score Model

**FIGURE 3:**
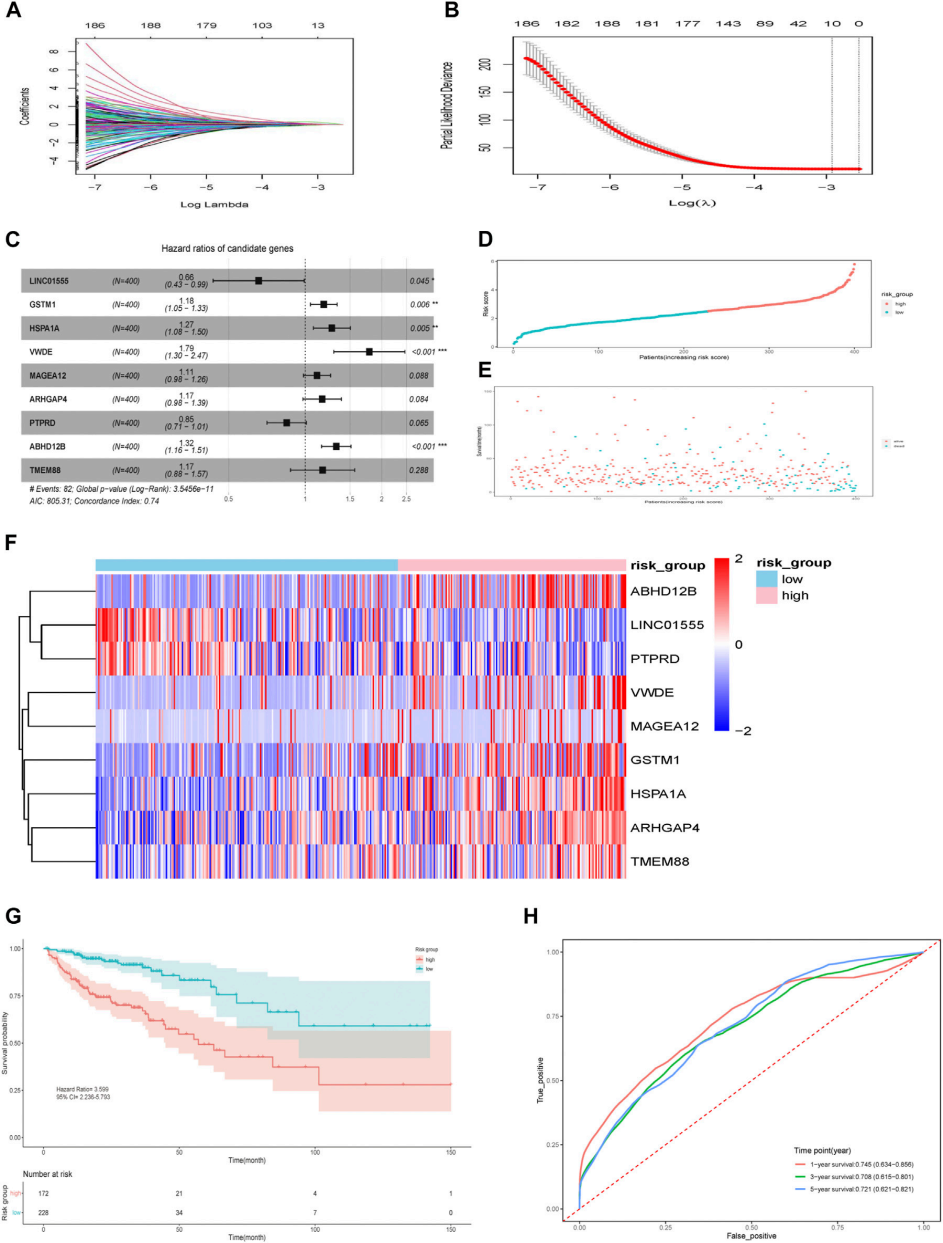
Identification of prognostic genes and nine-gene risk model construction in the TCGA cohort. **(A)** LASSO coefficients. **(B)** Plots of the cross-validation error rates. The dashes signify the value of the minimal error and greater λ value. **(C)** Multivariable Cox proportional hazard model of nine genes. **(D)** Risk score distribution in the two groups. **(E)** Survival overview in the two groups. **(F)** Heatmap of nine genes in the two groups. **(G)** Survival curve of the two groups. **(H)** Time-dependent ROC curve for 1-, 3-, and 5-years survival prediction. *p < 0.05, **p < 0.01, ***p < 0.001.

We included 400 patients with RNA-seq data and complete clinical information from the TCGA database for subsequent analysis. The patients’ characteristics are summarized in Table 1. Univariable Cox regression analysis was performed for 705 genes and 40 genes with *p* < 0.05 were selected. Besides, the same 705 genes were brought into LASSO regression analysis and 10 genes were selected (Figures 3A,B). The intersection of above two gene sets, including nine genes (*LINC01555*,*GSTM1*,*HSPA1A*, *VWDE*, *MAGEA12*,*ARHGAP4*,*PTPRD*, *ABHD12B*,*TMEM88*) were eventually screened out as prognosis-related genes. Supplementary Figure S2 showed the correlation between gene expression and corresponding methylation level of nine genes. Then, multivariable Cox regression analyses were performed, and a nine-gene model was constructed according to their expression levels and coefficients (Figure 3C). The formula was as follow: risk score = (−0.421*LINC01555)+(0.168*GSTM1)+(0.241*HSPA1A)+(0.581*VWDE)+(0.107*MAGEA12)+(0.153*ARHGAP4)+(−0.168*PTPRD)+(0.281*ABHD12B)+(0.157*TMEM88). Based on the formula, we calculated the risk score for each patient and used X-tile software to identify the optimal cut-off value (2.51). Those with a risk score over the cut-off value, 172 patients in total, were classified as the high-risk group, while the remaining 228 patients were classified as the low-risk group (Figure 3D). Intuitively, more people died in the high-risk group than in the low-risk group (Figure 3E). The expression profiles of nine genes in all patients and the corresponding risk group were presented in the form of heat map (Figure 3F). The Kaplan–Meier analysis of all patients demonstrated that the high-risk group had a significantly shorter OS (*P* = 2e-08) (Figure 3G). The AUC of this nine-gene risk assessment model was 0.745 (95% CI 0.634–0.856), 0.708 (95% CI 0.615–0.801) and 0.721 (95% CI 0.621–0.821) at 1-, 3- and 5- years, respectively (Figure 3H).

**TABLE 1:** Summary of the patients’ demographics and clinical characteristics.

Variables	Groups	Patients	
		Training set (*n* = 400)	Validation set (*n* = 550)
Age [n (%)]			
—	Median	68	68
—	Range	57.8–77.0	59.0–76.0
—	≤65 years	166 (41.5%)	219 (39.8%)
—	>65 years	234 (58.5%)	331 (60.2%)
Gender [n (%)]			
—	Male	214 (53.5%)	299 (54.4%)
—	Female	186 (46.5%)	251 (45.6%)
T stage [n (%)]			
—	1	9 (2.3%)	16 (2.9%)
—	2	69 (17.2%)	47 (8.5%)
—	3	275 (68.7%)	372 (67.7%)
—	4	47 (11.8%)	115 (20.9%)
Lymph node metastatic [n (%)]			
—	Yes	166 (41.5%)	241 (43.8%)
—	No	234 (58.5%)	309 (56.2%)
Distant metastatic [n (%)]			
—	Yes	57 (14.3%)	60 (10.9%)
—	No	343 (85.7%)	550 (89.1%)
TNM stage [n (%)]			
—	Ⅰ	70 (17.5%)	40 (7.2%)
—	Ⅱ	156 (39.0%)	257 (46.8%)
—	Ⅲ	117 (29.3%)	194 (35.3%)
—	Ⅳ	57 (14.2%)	59 (10.7%)
Vital status			
—	Alive	318 (85.1%)	374 (68%)
—	Dead	82 (14.9%)	176 (32%)
Median follow up (months)			
—	—	23	53

**FIGURE 4:**
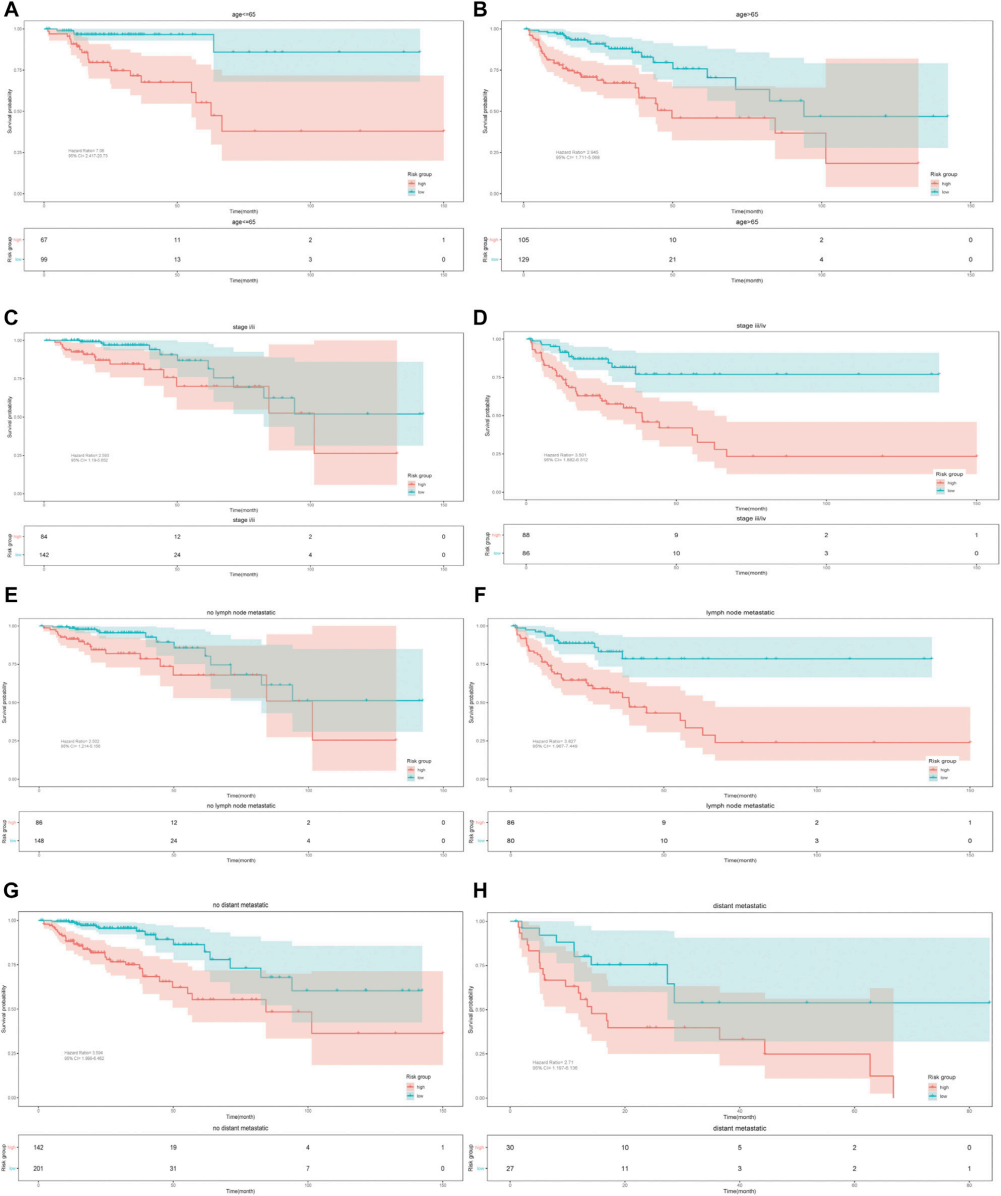
Kaplan–Meier survival curves. Validation of the nine-gene model based on different clinicopathologic characteristics.

We further tested the effect of the model in different subgroups divided by some clinicopathologic factors (age, TNM stage, lymph node metastatic and distant metastatic). The K-M survival analysis showed the same prediction trend in all subgroups, proving that the model has certain reliability and practicability in evaluating prognosis (Figure 4).

### Development and Evaluation of a Nomogram for OS Prediction

**FIGURE 5:**
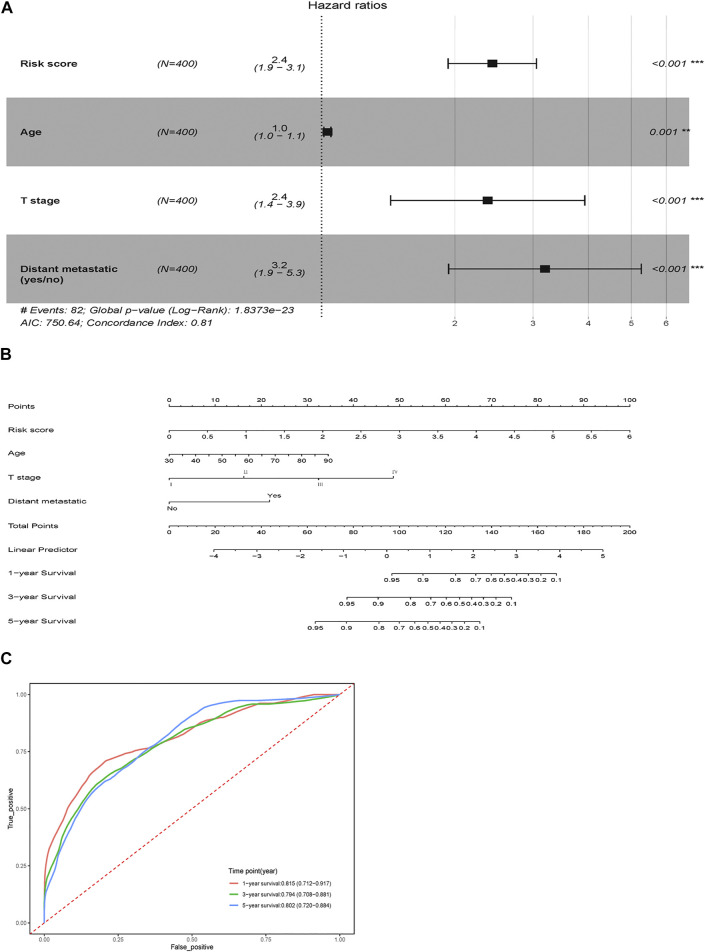
Nomogram for survival prediction. **(A)** Multivariable Cox proportional hazard model of the risk score and clinicopathologic factors. **(B)** OS-associated nomogram. **(C)** Time-dependent ROC curve for 1-, 3-, and 5-years survival prediction. **p < 0.01, ***p < 0.001.

Risk score, age, T stage, lymph node metastatic and distant metastatic were selected as significant predictive factors after univariable regression analysis (Table 2). Stepwise regression analysis showed that after removing the factor lymph node metastatic, AIC of multivariable Cox regression went down from 751.65 to 750.64, so it was further ruled out. Therefore, four independent risk factors (risk score, age, T stage and distant metastatic) were ultimately retained to build a visualized and applicable nomogram (Figures 5A,B). The C-index of the model is 0.81 (95% CI: 0.754–0.868). According to Nomogram, each variable was assigned a corresponding score, as a result we could calculate a total score for each patient. The AUC of nomogram was 0.815 (95% CI 0.712–0.917), 0.794 (95% CI 0.708–0.881) and 0.802 (95% CI 0.720–0.884) at 1-, 3- and 5- year, respectively (Figure 5C). The results revealed that the predicted survival possibility by the nomogram was close to the actual survival situation. Furthermore, our nomogram model performed better than the model only using significant clinicopathological factors (Supplementary Table S1).

**TABLE 2:** Univariable and multivariable Cox regression analyses in TCGA cohort.

	Univariable cox regression				Multivariable cox regression			
		95% CI				95% CI		
	HR	Lower	Upper	*p*	HR	Lower	Upper	*p*
Risk score	2.718	2.163	3.416	<0.001	2.429	1.931	3.055	<0.001
Age	1.019	1	1.039	0.045	1.031	1.012	1.05	0.001
Gender (male/female)	1.183	0.764	2.832	0.452	—	—	—	—
T stage	3.163	2.036	4.915	<0.001	2.372	1.432	3.929	<0.001
Lymph node metastatic (yes/no)	2.772	1.767	4.346	<0.001	—	—	—	—
Distant metastatic (yes/no)	4.965	3.133	7.868	<0.001	3.193	1.936	5.267	<0.001

### External Validation of the Nomogram

**FIGURE 6:**
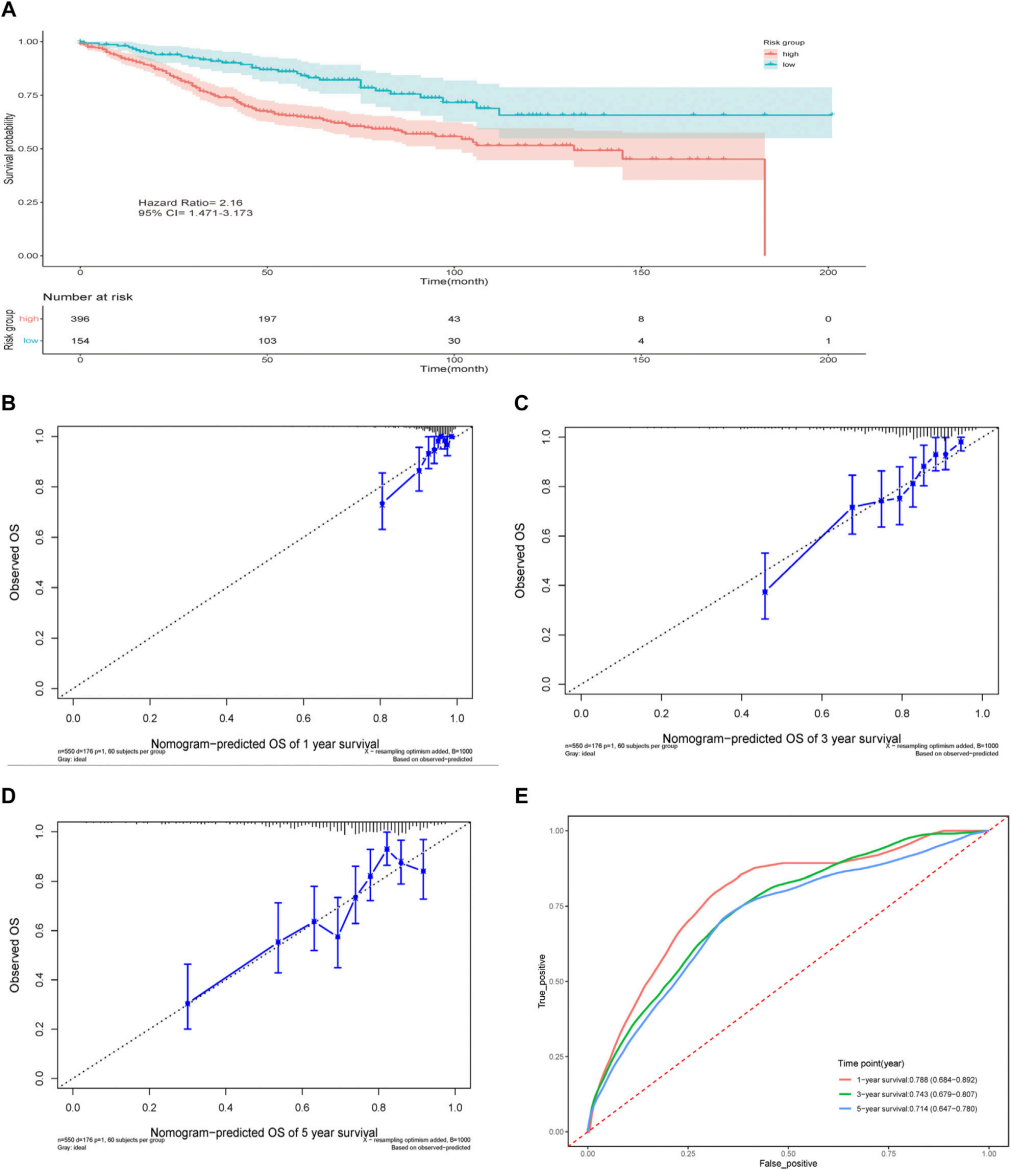
Validation of the prediction model. **(A)** Survival curve of the two groups. **(B–D)** Nomogram calibration plots at 1-, 3-, 5-years in the validation cohort. **(E)** Time-dependent ROC curve for 1-, 3-, and 5-years in the validation cohort.

The nomogram established above was further validated in the GEO dataset GSE39582 (Figures 6A–E). Totally 550 patients with complete basic clinical information were included for analysis (Table 1). The calibration curves for the predicted possibility of 1-, 3- and 5-years survival displayed obvious concordance between the predicted results and the actual observations in the GEO dataset (Figures 6B–D). The calibration slope was 1.56 (95% CI 1.31–1.82) at 1-year survival, 1.21 (95% CI 1.05–1.36) at 3-years survival and 1.00 (95% CI 0.74–1.26) at 5-years survival. Similar to the performance in TCGA cohort, the AUC of tdROC was 0.788 (95% CI 0.684–0.892) at 1-year survival, 0.743 (95% CI 0.679–0.807) at 3-years survival and 0.714 (95% CI 0.647–0.780) at 5-years survival (Figure 6E). The concordance index of the nomogram was 0.722 (95%CI 0.683–0.761). To sum up, the results showed that our model performed well in the validation cohort.

### Molecular Characteristic of Different Risk Groups

**FIGURE 7:**
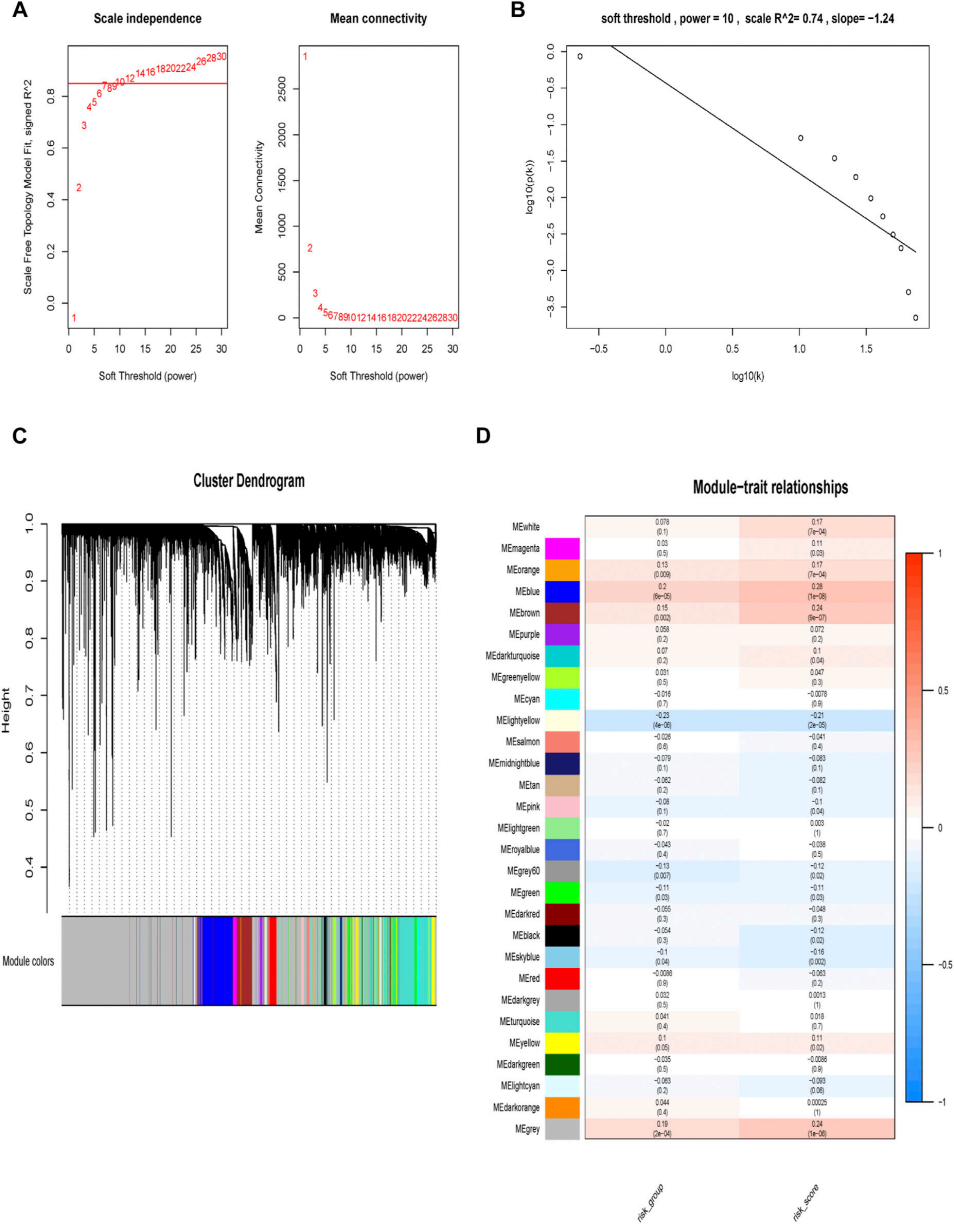
Construction of weighted gene correlation network. **(A, B)** Screening and validation of the soft threshold. **(C)** Clustering dendrogram of genes. **(D)** Correlation between modules and risk model and identification of the key module.

**FIGURE 8:**
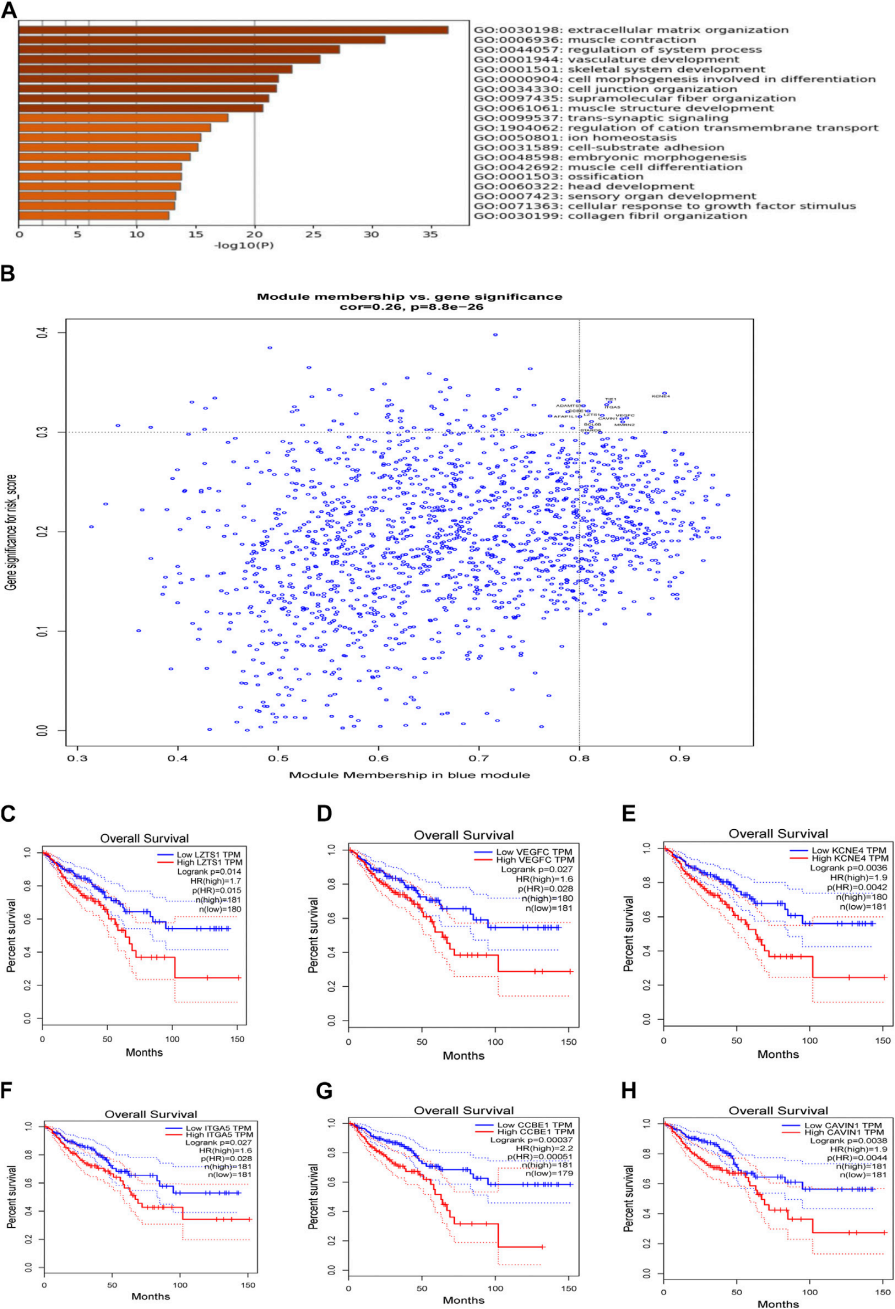
Analysis of weighted gene correlation network. **(A)** Enrichment analysis in the Metascape database and the top 20 enrichment terms were shown. **(B)** Identification of the hub genes (|MM|>0.8, |GS|>0.3). **(C)** Association of OS and LZTS1 expression in GEPIA database. **(D)** Association of OS and VEGFC expression in GEPIA database. **(E)** Association of OS and KCNE4 expression in GEPIA database. **(F)** Association of OS and ITGA5 expression in GEPIA database. **(G)** Association of OS and CCBE1 expression in GEPIA database. **(H)** Association of OS and CAVIN1 expression in GEPIA database.

We used two approaches to comprehensively explore molecular characteristic of different risk groups. Firstly, we performed WGCNA analysis in the TCGA cohort. In current study, the power of β = 10 (scale-free *R* ^2^ = 0.74) was selected as a soft threshold parameter to ensure a scale-free network (Figures 7A,B). The co-expression gene modules were then constructed and divided into 28 meaningful modules via the average linkage hierarchical clustering (Figure 7C). Blue module was found to have the highest association with 9-gene risk model (Cor = 0.28, *P* = 1e-08 for risk score; Cor = 0.2, *P* = 6e-05 for risk group) (Figure 7D). There were a total of 1,577 genes in the blue module. We then used Metascape to analyze these genes. Top 20 clusters of functional enriched sets were presented (Figure 8A). Based on the cut-off criteria (|MM|>0.8, |GS|>0.3), twelve hub genes (*LZTS1, TIE1, STARD8, VEGFC, KCNE4, ADAMTS1, AFAP1L1, ITGA5, BCL6B, MMRN2, CAVIN1* and *CCBE1*) with high connectivity in the clinical significant module were identified (Figure 8B). The expression of all twelve genes showed significant correlation with risk score (Supplementary Figures S3A–L). The hub genes were then analyzed in the GEPIA database. For *LZTS1, VEGFC, KCNE4, ITGA5, CCBE1* and *CAVIN1*, K-M survival analysis revealed that a higher expression was meaningfully associated with a worse prognosis (Figures 8C–H).

**FIGURE 9:**
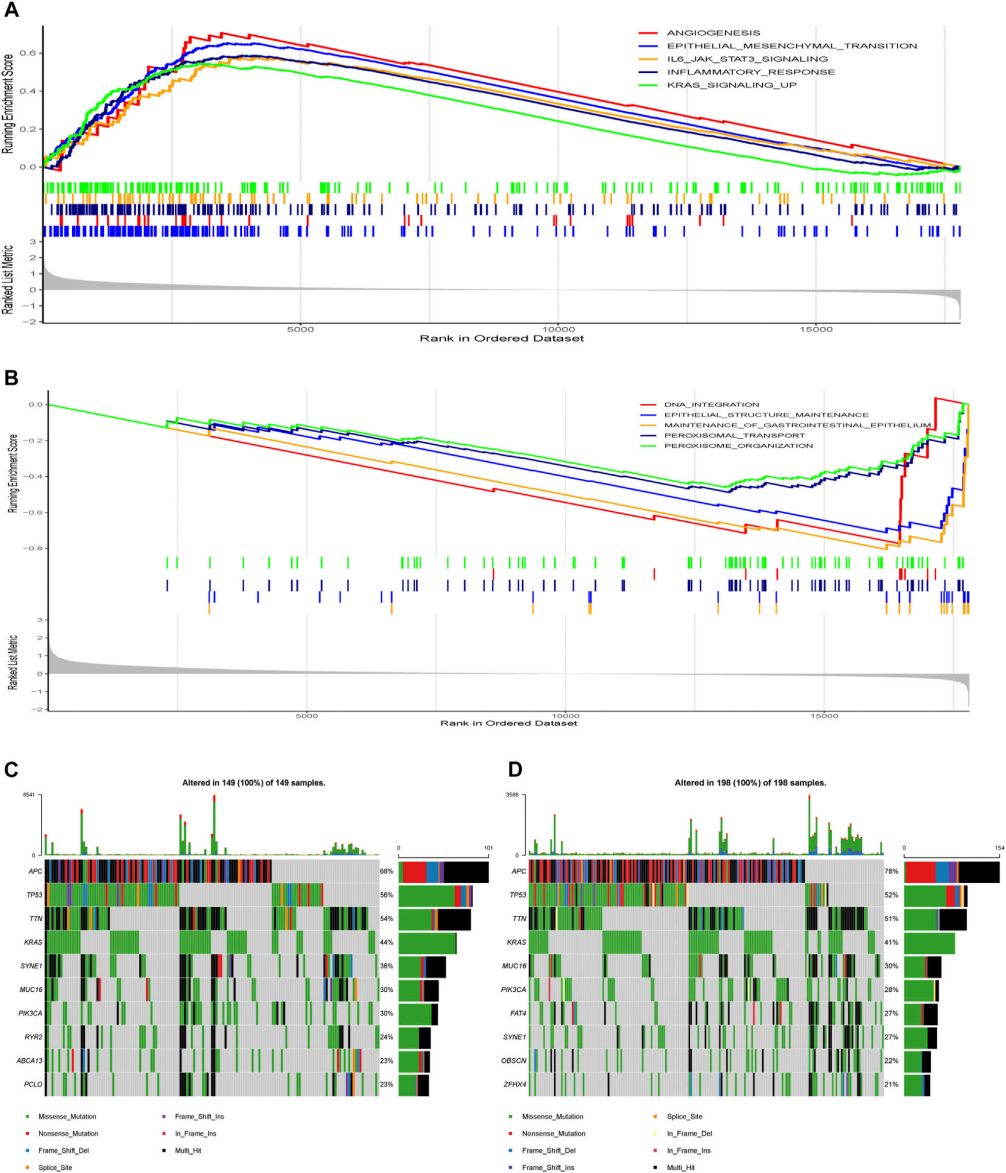
Molecular characteristics of different risk groups. **(A)** Gene sets enriched in high-risk group. **(B)** Gene sets enriched in low-risk group. **(C)** Significantly mutated genes in the mutated samples of high-risk group. **(D)** Significantly mutated genes in the mutated samples of low-risk group. Mutated genes (rows, top 10) are ordered by mutation rate. The right shows mutation percentage, and the top shows the overall number of mutations. The color-coding indicates the mutation type.

Besides, GSEA was performed in the two risk groups. A false discovery rate (FDR) less than 0.25 and an absolute value of the normalized enrichment score (NES) greater than 1 were defined as the cut-off criteria. The gene sets of the high-risk group were enriched in tumor progression and metastasis-related pathways and inflammatory response-related pathways (Figure 9A), while the gene sets of the low-risk group were enriched in DNA integration, epithelial structure maintenance related pathways (Figure 9B).

Next, we analyzed gene mutations to further understand different molecular subgroup associated with the nine-gene risk score (Figures 9C,D). The mutation rates of *APC*, *TP53*, *TTN*, *KRAS*, *SYNE1*, *MUC16* and *PIK3CA* were higher than 20% in both groups. The mutation of the *RYR2*, *ABCA13* and *PCLO* genes was more common in the high-risk group, while the mutation of *FAT4*, *OBSCN* and *ZFHX4* genes was more common in the low-risk group.

### Immune Characteristics of Different Risk Groups

**FIGURE 10:**
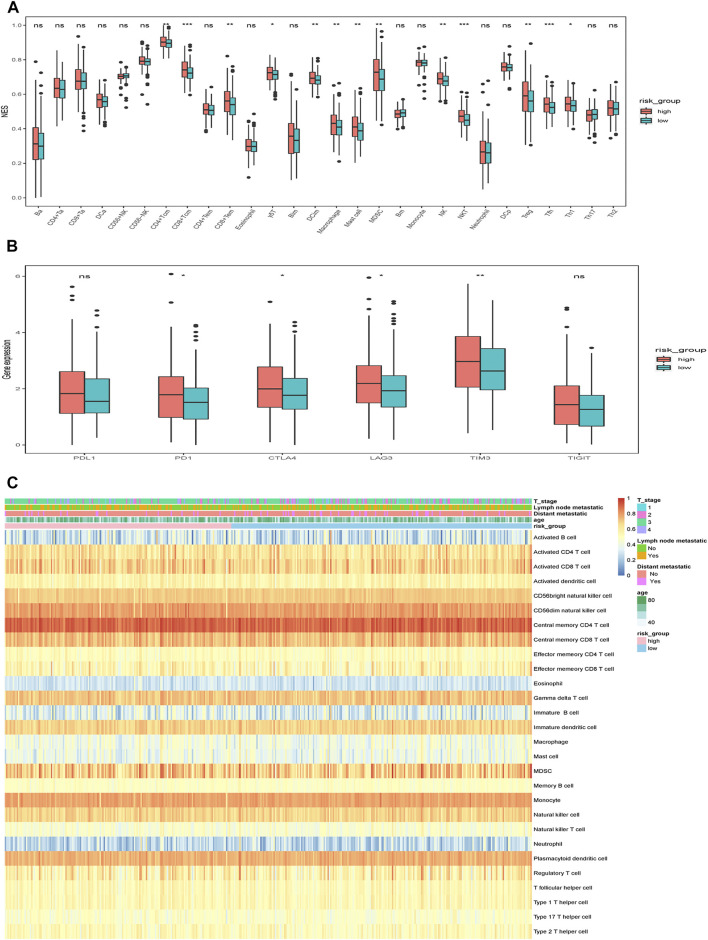
Immune characteristics of different risk groups. **(A)** The relative abundance of TIICs in different risk groups. **(B)** The expression of ICPs in different risk groups. The scattered dots represent the outliers of the two risk groups. The thick lines represent the median value. The bottom and top of the boxes are the 25th and 75th percentiles (interquartile range), respectively. Significant statistical differences between the two groups were assessed using the Wilcoxon test. **(C)** The relative abundance of TIICs for 400 patients in the TCGA cohort. ns: not significant, *p < 0.05, **p < 0.01, ***p < 0.001.

Given that the features of tumor-infiltrating immune cells (TIICs) are correlated with the development and progression of cancer and may influence the prognosis, including CRC, we then used ssGSEA method to analyze the infiltration of various immune cells in tumor samples. We found that cells executing pro-tumor suppression (such as MDSCs, regulatory T cells, immature dendritic cells) were more abundant in the high-risk group (Figure 10A). Additionally, the correlation between the risk and common ICPs, i.e., *PDCD1* (PD1), *CD274* (PDL1), *CTLA4*, *LAG3*, *HAVCR2* (TIM3) and *TIGIT*, was analyzed. Consistent with the higher infiltration of immunosuppressive cells in high-risk group, several common ICPs showed a significantly higher expression in the high-risk group (Figure 10B). The immune landscape and clinicopathological characteristics of different risk groups are presented as heat map in Figure 10C.

## Discussion

Pathological staging (tumor-node-metastases classification system) is a significant factor in clinical decision-making and prognosis evaluation of CRC. However, clinical outcomes often differ remarkably among patients at the same stage, suggesting that the current staging system is not sufficient in reflecting individual biological heterogeneity and predicting patient outcomes. A new prognostic assessment model referring to molecular and genetic profile may guide individualized therapy and improve long-term outcomes.

DNA methylation is an important mechanism in the regulation of gene expression. Aberrant methylation is frequently observed in tumors. These changes may promote malignant transformation by upregulating the expression of proto-oncogenes or inhibiting the expression of tumor suppressor genes. Abnormal methylation of some specific genes may be a key event in the early development of tumors and has potential to be a predictive biomarker for prognosis. With advances in sequencing technology, epigenetic changes of genes can be easily detected with a high degree of accuracy. Therefore, we adopted a beta mixture model-based method (MethylMix) to identify DNA methylation-driven genes. We identified 705 methylation-driven genes in CRC. Functional enrichment analysis revealed that these genes were involved in a wide range of biological processes and pathways, including cell signal transduction, cell differentiation, apoptosis regulation, metabolism, etc. These results suggest that DNA methylation is involved in gene dysregulation influencing various physiological processes and imply a possible mechanism by which DNA methylation is functionally associated with progression and prognosis in patients with CRC.

In our study, combining LASSO regression and multivariable Cox regression analysis, nine genes that were closely related to survival and prognosis were selected. Most of these genes have been reported in cancer studies. Glutathione S-transferase Mu 1 (*GSTM1*), a member of the glutathione S-transferase family, functions primarily as a detoxification enzyme, also involved in the negative regulation of apoptosis-related signaling pathways (Kalinina et al., 2014). Aberrant methylation of GSTM1 has been found in various cancers, such as acute myeloid leukaemia, urothelial carcinoma and head and neck cancer (Sharma et al., 2010; Ohgami et al., 2012; Wang et al., 2016). However, the methylation changes in CRC remain to be further validated. Heat shock 70 kDa protein 1A (*HSPA1A*), a major member of the 70 kDa stress protein family, is increased in a variety of tumor types (Calderwood et al., 2006). High level of intracellular HSPA1A can prevent cancer cells from apoptotic cell death, promote cancer cells proliferation or migration, and mediate therapeutic resistance, thus contributing to the formation of aggressive tumor phenotypes (Shevtsov et al., 2018). Melanoma-associated antigen 12 (*MAGEA12*) is also highly expressed in some tumor cells and has been found to exert oncogenic role by promoting the ubiquitination and degradation of the tumor suppressor p21 (Yanagi et al., 2017). Besides, DNA hypomethylation of MAGEA12 has been identified as a candidate biomarker for liver cancer diagnosis and prognosis (Stefanska et al., 2013). Rho GTPase-activating protein 4 (*ARHGAP4*) belongs to the small GTPase family, which can hydrolyze the active GTP into inactive GDP and negatively regulate RhoA protein. Studies showed that it was involved in regulating the invasion and metastasis of pancreatic cancer cells (Shen et al., 2019a; Shen et al., 2019b). Receptor-type tyrosine-protein phosphatase delta (*PTPRD*) is frequently found to be inactivated by epigenetic modification in a variety of tumors, suggesting that it may have tumor suppressive effects (Kohno et al., 2010; Funato et al., 2011; Giefing et al., 2011). Based on methylation-specific PCR, frequently hypermethylation of PTPRD promoter has been validated in CRC, which may mediate the gene inactivation (Mokarram et al., 2009). Furthermore, *in vitro* experiments also showed that exogenous expression of PTPRD could inhibit the migration and invasion of colon cancer cells (Funato et al., 2011). Transmembrane protein 88 (*TMEM88*), a newly discovered protein that is localized on cell membranes, can inhibit the classical Wnt signaling pathway and is thought to have a bidirectional effect of inhibiting or promoting cancer in different contexts (Ge et al., 2018). Indeed, promoter hypermethylation of TMEM88 is associated with poorer prognosis of non-small cell lung cancer (Ma et al., 2017), whereas the hypomethylation is associated with platinum resistance in ovarian cancer (de Leon et al., 2016). What effect of aberrant TMEM88 methylation has on CRC needs to be further explored. LINC01555 is a long non-coding RNA that has rarely been studied. Von Willebrand factor D and EGF domain-containing protein (*VWDE*) is a blastema-enriched gene in a variety of highly regenerative species (Leigh et al., 2020). A recent study showed that VWDE is a significant driver oncogene with a highly mutation prevalence in breast cancer (Pongor et al., 2015). Abhydrolase domain containing 12B (*ABHD12B*) locates on chromosome 14. Physiological and pathological functions of ABHD12B are rarely reported. Given the significant association of these three genes (*LINC01555*, *VWDE* and *ABHD12B*) with prognostic in CRC, it is meaningful to further study that how they regulate the development of CRC.

Furthermore, based on the expression of these nine genes and coefficients with survival, a prognostic model was established. Each patient’s risk score was calculated and all patients were divided into two groups based on the X-tile software. The K-M survival curve revealed that the model could distinguish the difference of OS between the two risk groups. Time-dependent ROC analysis showed that the nine-gene risk model had a good performance in predicting OS of CRC. Our risk model was a promising prognostic biomarker for CRC patients.

To determine the molecular mechanisms which drive the nine gene risk model, we took many approaches. Firstly, WGCNA was performed to explore clinical significant modules associated with the nine-gene signature. The blue module, which contained 1,577 genes, was found to have the highest association with risk model. GO/KEGG enrichment analysis by Metascape showed that the functions of “extracellular matrix organization,” “vasculature development,” “cell junction organization,” “cell-substrate adhesion,” “collagen fibril organization” were significantly enriched. Many of these pathways have been reported to be involved in tumor onset and development. According to the cut-off criteria |MM| > 0.8, |GS| > 0.3, twelve genes with a high connectivity were screened out from the clinical significant module. The analysis using GEPIA also confirmed that high expression of *LZTS1, VEGFC, KCNE4, ITGA5, CCBE1 and CAVIN1* was significantly associated with poorer prognosis in CRC. Leucine zipper putative tumor suppressor 1 (*LZTS1*) was reported to suppress cancer cell growth and regulate cell cycle (Ishii et al., 2001). However, given that the higher expression was associated with shorter OS, the specific role of LZTS1 in CRC needs further research. Vascular endothelial growth factor C (*VEGFC*) is active in blood vessels and lymphatics endothelial cell proliferation and migration, therefore contributing to tumor metastases (Tacconi et al., 2015). Recently, VEGFR3 was found to be expressed in tumor-associated macrophages (TAMs) in CRC. VEGFC/VEGFR3 pathway could inhibit antitumor immunity by regulating TAMs (Tacconi et al., 2019). Potassium voltage-gated channel subfamily E member 4 (*KCNE4*) has been found to be increased in multiple solid tumors (Biasiotta et al., 2016). Some studies also show a direct association between the transmembrane ion transport and carcinogenesis, although the exact mechanism is still unclear (Cone, 1969). Integrin alpha-5 (ITGA5) can form heterodimer with different beta subunits. Down-regulation of ITGA5 in CRC cells could inhibit cell proliferation and tumorigenesis and promote cell apoptosis (Yu et al., 2019). Besides, one study shows that ITGA5 is required for the tumor supportive role of fibroblasts (Lu et al., 2019). Collagen and calcium-binding EGF domain-containing protein 1 (*CCBE1*) is important for lymphatic vascular development and plays a pro-tumor role in colorectal cancer by promoting lymphangiogenesis and lymphatic metastasis of cancer cells (Song et al., 2020). Indeed, in the TCGA cohort CCBE1 expression was significantly higher in patients with lymph node metastasis compared to those without lymph node metastasis (Supplementary Figure S4A). Caveolae-associated protein 1 (*CAVIN1*) cooperates with Caveolin to regulate lipid uptake of cell. Cavin1 can promote the secretion of extracellular vesicles (EVs) in glioma, and EVs expressing cavin1 in turn promote the growth of glioma (Wang et al., 2020).

We also used GSEA to analyze the differential gene expression between the high-risk group and the low-risk group. Consistent with the results of WGCNA, the biological processes or pathways enriched in the high-risk group were mostly involved in tumor invasion and progression. For example, activation of the transcription factor STAT3 has been widely reported in various tumors, including CRC (Bollrath et al., 2009). Phosphorylation and nuclear translocation of STAT3 drive transcription of genes involved in cell-cycle regulation, cell survival, cell migration, ultimately promoting invasion and metastasis of tumors (Yu et al., 2014).

Numerous studies have revealed that the features of tumor-infiltrating immune cells (TIICs) are closely correlated with the development and progression of cancer (Grivennikov et al., 2010; Steidl et al., 2010; Li et al., 2011). Understanding the landscape of the TIICs could help in finding new strategies to treat CRC. In our report, there were obvious differences in tumor immune cell composition between high-risk and low-risk groups. Notably, some immunosuppressive cells (MDSCs, regulatory T cells, immature dendritic cells), which might favor tumor growth (Jia et al., 2018), were more enriched in the high-risk group. Besides, the proportion of TAMs was significantly higher in the high-risk group than in the low-risk group. TAM infiltration is an independent prognostic risk factor for several kinds of cancer, including CRC (Steidl et al., 2010; Herrera et al., 2013; Waniczek et al., 2017; Ye et al., 2019). Accordingly, the expression of several common ICPs was higher in the high-risk group, further revealing that the high-risk group was characteristics of immunosuppression.

The efficacy of every single biomarker is inadequate. To further enhance the potential clinical application of this risk model, we established a nomogram. Nomogram combined multiple risk factors (nine-gene signature, age, T stage and distant metastatic) and constructed a simple statistical prediction model. Time-dependent ROC and calibration curve both showed that the predicted survival rate was close to the actual situation. Our nomogram might serve as an excellent predictive model in the prognosis assessment of CRC patients.

To the best of our knowledge, this nine-gene risk model has not been previously reported, and the information of DNA methylation driven genes provides a foundation for further exploration of the pathogenesis and therapeutic strategy of CRC. However, there are some limitations in current study. The risk model requires further confirmation by increasing the sample size and performing prospective, multicenter studies.

## Conclusion

In summary, our study established a nomogram that combined the DNA methylation-driven genes and some significant clinicopathological features. This model has been validated in different study cohorts and is expected to serve as a predictive tool with high sensitivity and specificity to assess clinical prognosis and to guide individualized anti-tumor therapy for patients with CRC.

## Data Availability

Publicly available datasets were analyzed in this study. This data can be found here: GSE39582.

## Abbreviations

AUC, area under the curve; CRC, colorectal cancer; DEGs, differentially expressed genes; GEPIA, gene expression profiling interactive analysis database; GSEA, gene Set enrichment analysis; FDR, false discovery rate; ICPs, inhibitory checkpoint molecules; MDSCs, myeloid-derived suppressor cells; NES, normalized enrichment score; OS, overall survival; ROC, receiver operating characteristic curve; TIICs, tumor-infiltrating immune cells; TAMs, tumor-associated macrophages; WGCNA, weighted gene correlation network analysis.
