# Supplementary material for: A Prognostic Model Based on Nine DNA Methylation-Driven Genes Predicts Overall Survival for Colorectal Cancer
Source: Front Genet. 2022 Jan 21;12:779383. doi: 10.3389/fgene.2021.779383 (PMC8814658; doi:10.3389/fgene.2021.779383)
Supplement: Supplementary file 1 [file DataSheet1.docx]

Supplementary Material

# Supplementary Figures and Tables

## Supplementary Figures


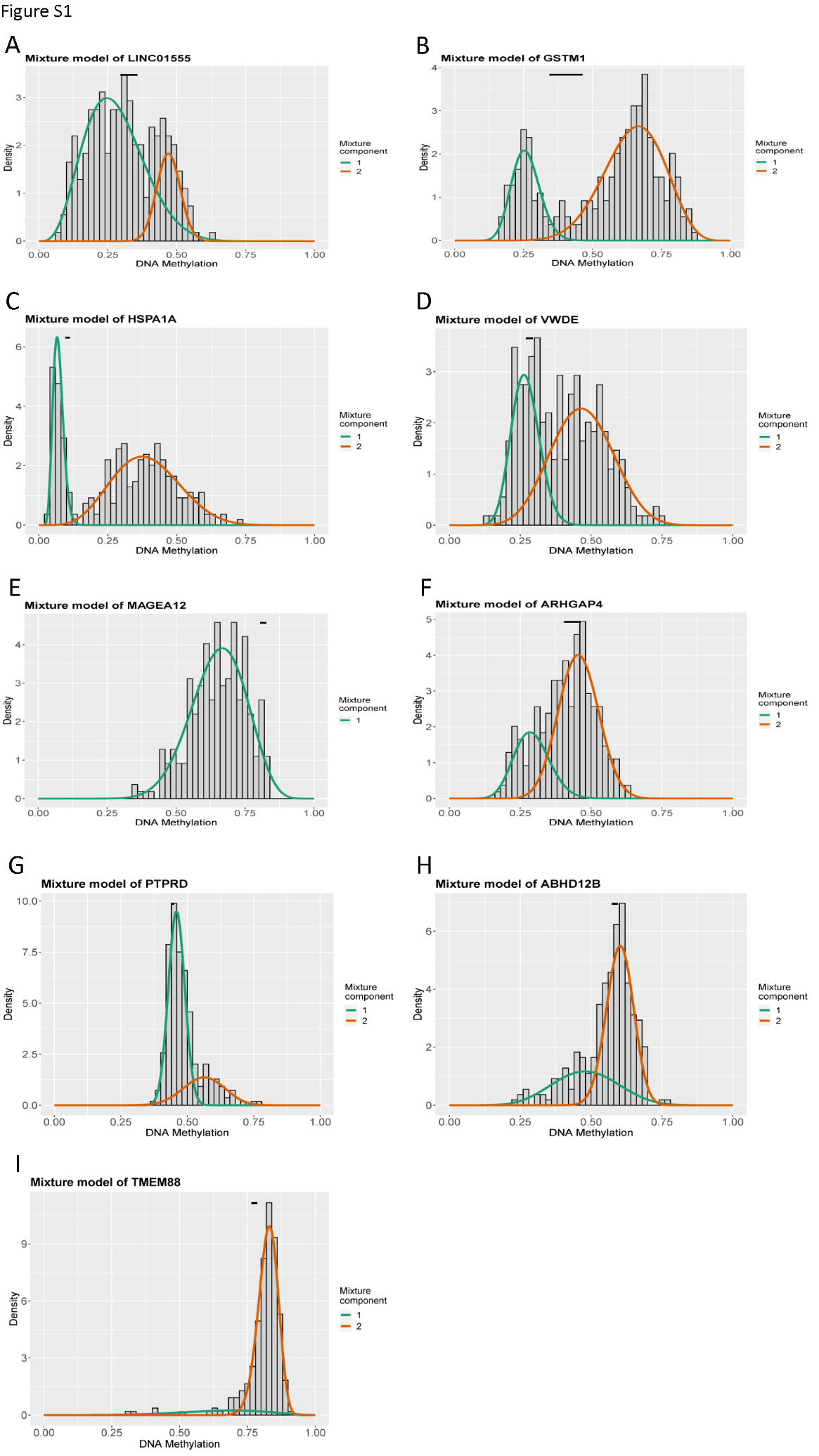


### Supplementary Figure 1.

**(A-I)** Mixture models of 9 of the 705 genes. The x-axis indicates the degree of methylation, the y-axis indicates the proportion at different degrees, the curve indicates the peak value, and the black bar indicates the normal methylation degree.


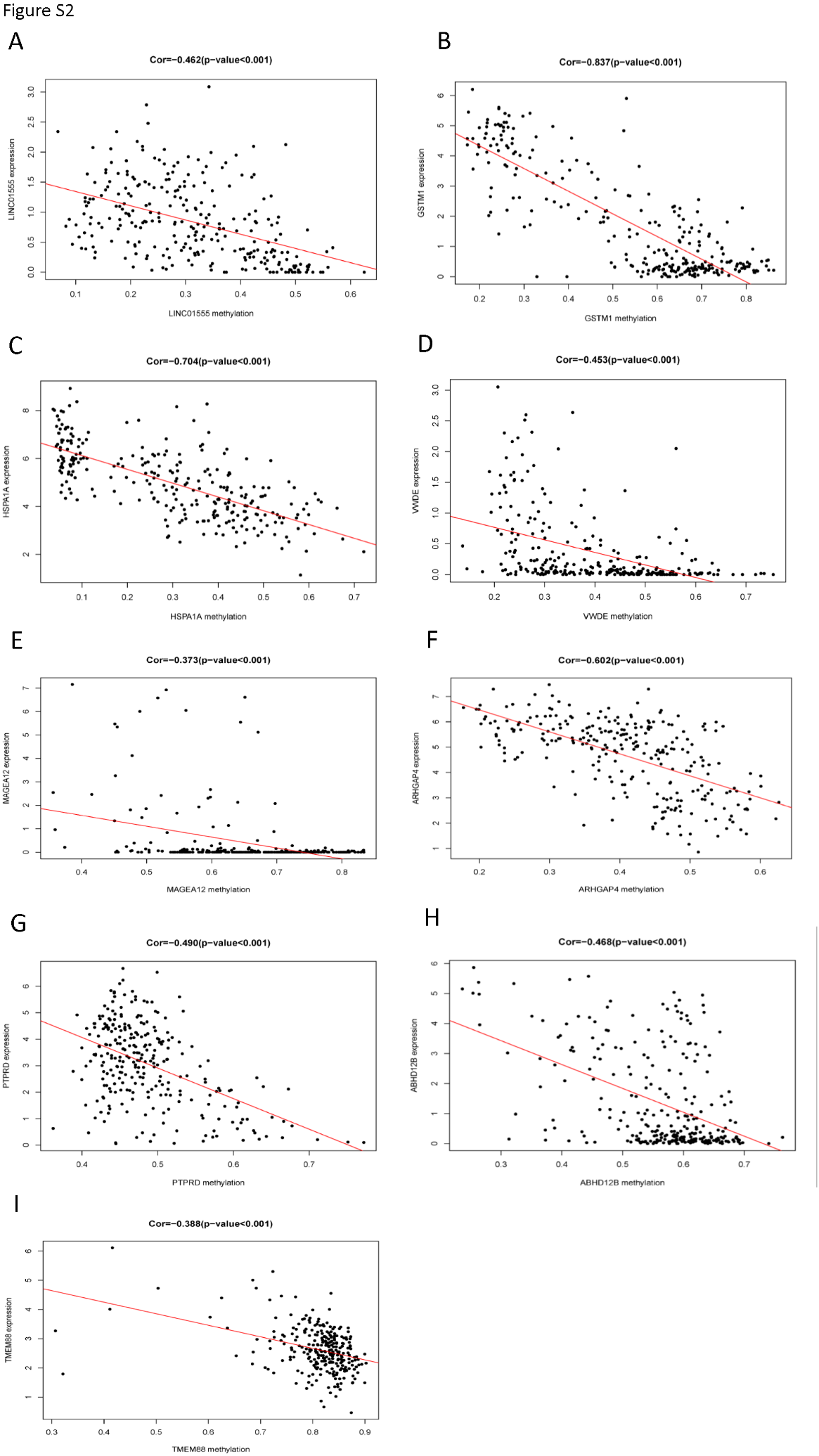


### 1.1.2 Supplementary Figure 2.

**(A-I)** Correlation analysis between the gene expression level and DNA methylation level of the nine genes. The vertical axis and the horizontal axis denote the gene expression and DNA methylation level, respectively.


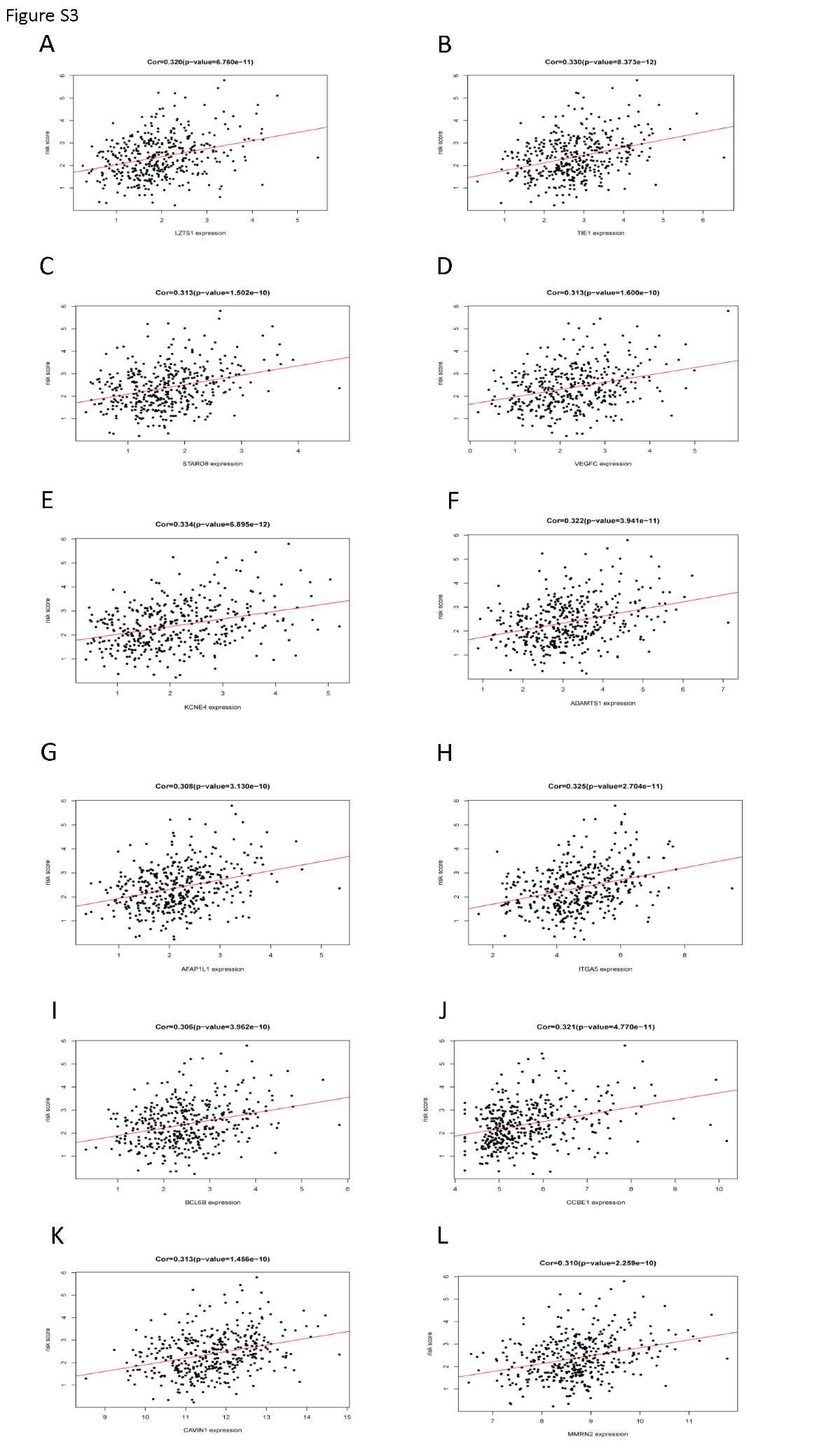


### Supplementary Figure 3.

**(A-L)** Correlation analysis between risk score and gene expression of twelve hub genes (*LZTS1, TIE1, STARD8, VEGFC, KCNE4, ADAMTS1, AFAP1L1, ITGA5, BCL6B, MMRN2, CAVIN1* and *CCBE1*). The vertical axis and the horizontal axis denote the risk score and gene expression, respectively.


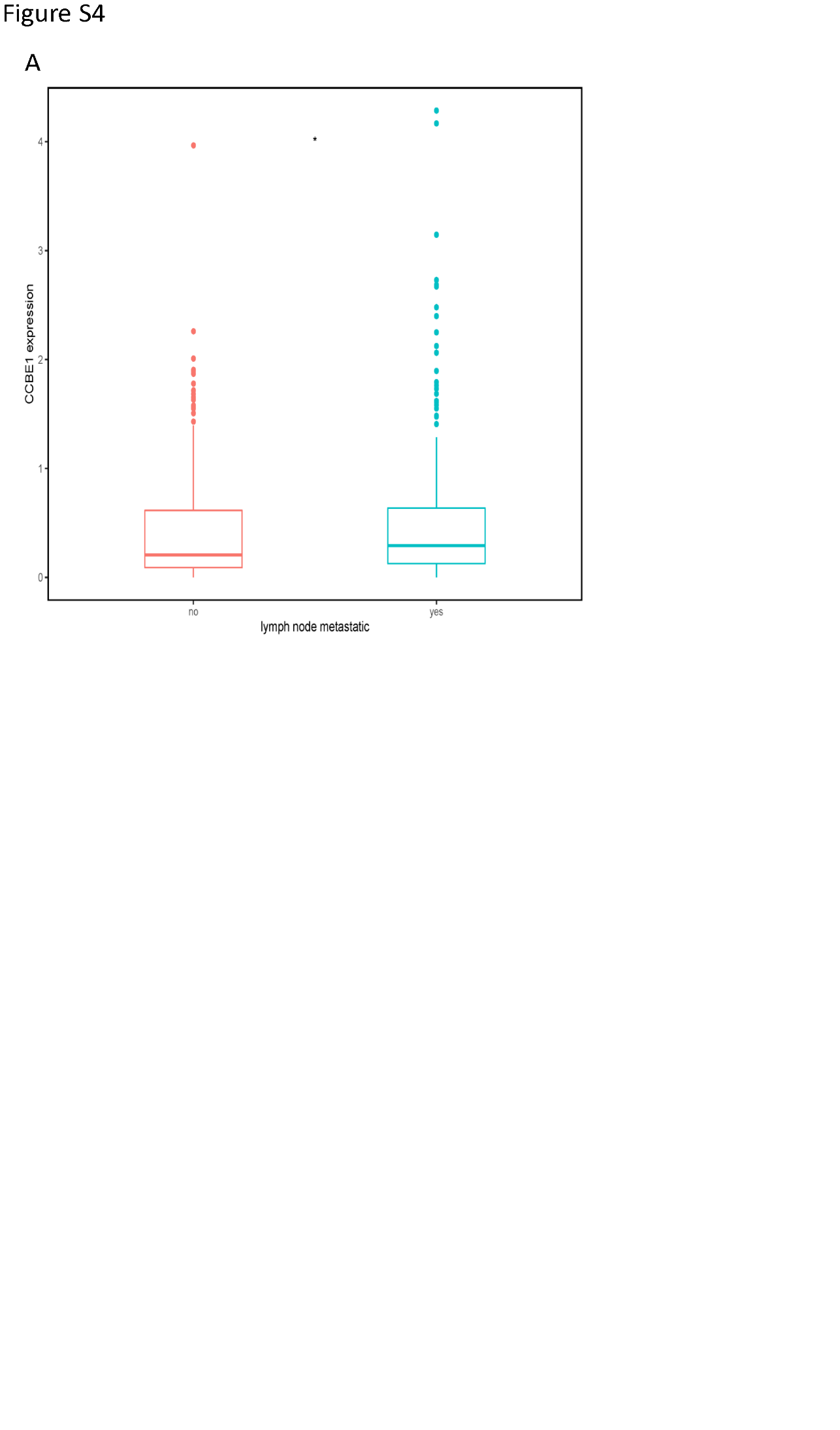


### Supplementary Figure 4.

(**A**) The association of gene expression of CCBE1 and lymph node metastatic in TCGA cohort.

## Supplementary Tables

### Supplementary Table 1 Comparison between nomogram and a model only using clinicopathological factors in TCGA cohort.

|  | C-index (95% CI) | calibration-slope (1-year) | calibration-slope (3-year) | calibration-slope (5-year) | AIC |
| --- | --- | --- | --- | --- | --- |
| Model 1 (combining nine-gene risk score and clinicopathological factors) | 0.811 (0.754-0.868) | 1.064 | 1.064 | 0.995 | 750.64 |
| Model 2 (only clinicopathological factors) | 0.74 (0.675-0.805) | 1.202 | 0.948 | 0.759 | 802.65 |
